# Molecular Interpretation of ACTH-β-Endorphin Coaggregation: Relevance to Secretory Granule Biogenesis

**DOI:** 10.1371/journal.pone.0031924

**Published:** 2012-03-05

**Authors:** Srivastav Ranganathan, Pradeep K. Singh, Uday Singh, Praful S. Singru, Ranjith Padinhateeri, Samir K. Maji

**Affiliations:** 1 Department of Biosciences and Bioengineering, Wadhwani Research Centre for Biosciences and Bioengineering, Indian Institute of Technology Bombay, Mumbai, India; 2 School of Biological Sciences, National Institute of Science Education and Research (NISER), Bhubaneswar, India; University of South Florida College of Medicine, United States of America

## Abstract

Peptide/protein hormones could be stored as non-toxic amyloid-like structures in pituitary secretory granules. ACTH and β-endorphin are two of the important peptide hormones that get co-stored in the pituitary secretory granules. Here, we study molecular interactions between ACTH and β-endorphin and their colocalization in the form of amyloid aggregates. Although ACTH is known to be a part of ACTH-β-endorphin aggregate, ACTH alone cannot aggregate into amyloid under various plausible conditions. Using all atom molecular dynamics simulation we investigate the early molecular interaction events in the ACTH-β-endorphin system, β-endorphin-only system and ACTH-only system. We find that β-endorphin and ACTH formed an interacting unit, whereas negligible interactions were observed between ACTH molecules in ACTH-only system. Our data suggest that ACTH is not only involved in interaction with β-endorphin but also enhances the stability of mixed oligomers of the entire system.

## Introduction

Amyloids are highly ordered protein/peptide aggregates that are associated with a number of human diseases including Alzheimer's and Parkinson's [1]. Natively structured or unstructured peptides/proteins undergo structural transition to form cross-β-sheet rich amyloids. This transition is known to happen via partially folded intermediates and soluble oligomers [2]. Many proteins/peptides can form amyloids under certain environmental conditions suggesting that amyloid formation is a generic property of polypeptide chains [3]. Several studies in the recent past have found that amyloids are also involved in several native biological functions [4], [5]. For example, amyloids of curli protein help *E. coli* for surface attachment and colonization [6]. Amyloids of chaplins are involved in aerial hyphae and fruiting body formation in filamentous fungi [7], [8]. Moreover, yeast prions [9], [10] and HET-s amyloid in *Podospora anserina* [11] are also well-studied examples of functional amyloids that are important for survival of the respective host and not just related to prevalence in diseased condition. The amyloid form of pmel17 in the melanosome appears to be used as a template for melanin synthesis and therefore protect melanocytes from reactive oxygen and free radicals [12]. In addition, aggregation has also been shown to be involved in secretory process of protein/peptide hormones [13].

Protein secretion in secretory cells of eukaryotes is primarily of two types: constitutive secretion and regulated secretion [14], [15]. In constitutive secretion, the newly synthesized proteins are secreted via vesicular trafficking immediately after exiting the Golgi without any external stimulus. Whereas, in regulated secretion, secretory proteins/peptides are stored for extended periods of time in a highly concentrated form within membrane-enclosed structures (secretory granules) in the cytoplasm [16], [17]. The secretory granules (SG) primarily consist of condensed aggregates of proteins/peptides [17], [18]. Many secretory proteins have been shown to form intermolecular aggregates at granule relevant conditions *in vitro*, and protein aggregates were also observed *in vivo*. These observations suggest that reversible aggregation is the necessary step for condensing and sorting of the secretory granule proteins (for review see; [17], [18], [19]. However, it was not clear whether the aggregated and concentrated proteins inside the granules are amorphous or contain any specific structure. It has recently been suggested that protein/peptides are stored as amyloid-like structures inside the secretory granules of pituitary [13]. A large number of secretory protein/peptide hormones formed non-toxic amyloids *in vitro* under granule relevant conditions. Also evident were amyloid like structures in the SG of hormones in AtT20 cells as well as rat pituitary tissue. However, the studies also showed that some of the peptide hormones such as ACTH and ghrelins do not form amyloids *in vitro*. Interestingly, they co-aggregated and formed amyloids in presence of β-endorphin (β-end) and obestatin, respectively [13]. ACTHs are also co-localized with β-end in the secretory granules of AtT20 cells. This study supported the hypothesis that heterotypic aggregation might be responsible for concentrating and sorting of non-aggregating peptide/protein hormone.

To understand the role of heterotypic aggregation between ACTH and β-end for their storage in secretory granules, we have performed experiments *in silico, in vitro* and *in vivo* and the results are presented in this paper. Our results suggest that unlike β-end, ACTH is neither able to change its conformation nor does it aggregate into amyloids *in vitro* (for the amino acid sequences see Table S1). Interestingly, ACTH and β-end co-localize in rat pituitary tissue, consistent with previous study suggesting that ACTH might require β-end as its aggregation partner [13]. To delineate the molecular interactions and possible early events of oligomerization of ACTH and β-end, we performed all atom molecular dynamics (MD) simulations in explicit solvent. The data suggest that ACTH is able to interact with β-end to form mixed trimers similar to the β-end-only simulation. We also find that, in presence of β-end, ACTH not only participates in oligomerization, but also enhances the overall stability of the mixed trimer. In contrast, significant intermolecular interactions were absent in ACTH-only simulation, suggesting that ACTH requires β-end for its conformational transition and aggregation in secretory granules. Furthermore, we performed aggregation studies on full-length β-end and N-terminal truncated β-end (β-end (6–31)) *in vitro*; the data suggested that N-terminal residues play a role in β-end aggregation as seen in our MD simulations.

## Methods

### *In vitro* aggregation of ACTH and β-end

Human ACTH (hACTH), human β-end (hβ-end) and β-end (6–31) were purchased from BACHEM. Peptides were dissolved either in 5% D-mannitol, pH 5.5 or in PBS, 0.01% sodium azide at a concentration of 2 mg/ml in 1.5 ml eppendorf tubes. The aggregation study in presence of different concentrations of low molecular weight (LMW) heparin was initiated by mixing 10 mM of heparin solutions and 1 mM of ACTH solution in 5% D-mannitol. The eppendorf tubes containing hormone solutions were placed into an Echo Therm model RT11 rotating mixture (Torrey Pines Scientific) with a speed corresponding 50 r.p.m. inside a 37°C incubator. The fibril formation was monitored by circular dichroism (CD) and ThT binding studies. At the end of aggregation, electron microscopy (EM)/atomic force microscopy (AFM) studies were performed for morphological characterization. Three independent experiments were performed for each sample.

### Circular dichroism spectroscopy (CD)

10 µl of hormone solutions were diluted in 5% D-mannitol to 200 µl. The peptide solutions were placed into a 0.1 cm path-length quartz cell (Hellma, Forest Hills, NY). Spectra were acquired using a JASCO 810 instrument. All measurements were done at 25°C. Spectra were generally recorded over the wavelength range of 198–260 nm. For better elucidation of the differences in secondary structure, the CD data of full-length β-end in presence of heparin at day 4 is represented from 260 nm to 207 nm. Three independent experiments were performed with each sample. Raw data were processed by smoothing and subtraction of buffer spectra, according to the manufacturer's instructions.

### Thioflavin T (ThT) binding

A 10 µl aliquot of peptide sample was diluted to 500 µl 5% D-mannitol containing 0.01% (w/v) sodium azide. The solution was mixed with 2 µl of 1 mM ThT prepared in the same solution. Fluorescence was measured immediately after addition of ThT using 10 mm rectangular quartz micro-cuvette. The fluorescence was measured on a spectrofluorimeter with excitation at 450 nm and emission at 482 nm or between 460–500 nm. The fluorescence intensity at 482 nm was plotted in the graph. Three independent experiments were performed for each sample.

### Electron microscopy (EM)

5 µl aliquot of peptide samples were diluted into 50 µl of water to a peptide concentration of ∼40 µM, spotted on a glow-discharged, carbon-coated Formvar copper grid (Electron Microscopy Sciences, Fort Washington, PA), incubated for 5 min, washed with distilled water, and then stained with 1% (w/v) aqueous uranyl formate solution. Uranyl formate solutions were filtered through 0.2 µm sterile syringe filters (Corning) before use. EM analysis was performed using a FEI Tecnai G2 12 electron microscope at 120 KV with nominal magnification between 26,000 to 60,000. Images were recorded digitally by using the SIS Megaview III imaging system. At least two independent experiments were carried out for each sample.

### Atomic force microscopy (AFM)

For AFM, 2 mg/ml peptide solutions were diluted into 100 fold by water and diluted sample was spotted on a freshly cleaved mica sheet followed by washing with water. The mica was dried under vacuum desiccators. The imaging was done in tapping mode under a silicon nitride AFM cantilever using Veeco Nanoscope IV multimode AFM. At least five different areas of two independent samples were scanned with a scan rate of 1.0 Hz.

### 2,2,2-Trifluoroethanol (TFE) titration of ACTH and β-end

TFE titration of ACTH and β-end was carried out to probe the intrinsic ability of these peptides to form ordered structure. To do so, ACTH and β-end were dissolved at a concentration of 2 mg/ml in phosphate buffered saline (pH 7.4). The peptide solutions and TFE were mixed such that the resulting concentration of ACTH and β-end is of 45 µM and TFE was varied from 0 to 80% (V/V). The resulting solution was used immediately for CD measurement as described before.

### Immunohistochemistry

Adult, male, Wistar rats weighing 220–250 g were housed under standard environmental condition (light between 600 and 1800 h, temperature 22±1°C, chow and water *ad libitum*). All the experimental procedures were approved by the Institutional Animal Ethics Committee (Approval number 92/1999/CPCSEA), Department of Pharmaceutical Sciences, Nagpur University, Nagpur, India. Animals were anaesthetized with sodium pentobarbital and perfused transcardially with 20 ml phosphate buffered saline (PBS, 0.01 M, pH 7.4) followed by 150 ml 4% paraformaldehyde in phosphate buffer (0.1 M, pH 7.4). The pituitary glands were dissected out and postfixed in the same fixative overnight at 4°C. The pituitary glands were cryoprotected in 25% sucrose solution in PBS overnight at 4°C, frozen in mounting media (Tissue-Tek, Torrance, CA), sectioned on a cryostat (CM 1850, Leica) at 12 µm thickness in coronal plane and collected in PBS to get four series of alternate sections. Sections were stored at −20°C in freezing solution until processed further. Sections were rinsed in PBS and treated with 0.5% Triton X-100 in PBS for 20 min. The sections were incubated in 5% normal horse serum in PBS for 30 min and incubated overnight at 4°C in a mixture of anti-rabbit ACTH polyclonal antiserum (National Hormone Pituitary Program, NIH, USA) diluted 1∶2000 and rat monoclonal β-end antibody (Cat# ab54205, Abcam, USA) diluted 1∶2000 in antibody diluent. Sections were rinsed in PBS and incubated in a mixture of DyLight488-conjugated goat anti-rabbit IgG (Jackson Immunoresearch, USA, 1∶250) and Cy3-conjugated donkey anti-rat IgG (Jackson Immonoresearch, 1∶250) for 4 h at room temperature. Sections were rinsed in PBS followed by Tris (pH 7.6) and mounted with Vectashield mounting medium containing DAPI (Vector Laboratories, USA). Sections were observed under Olympus BX51 epifluorescence microscope using a dual filter set for DyLight 488 and Cy3 (Cy3, excitation 540–590 nm and emission 600–660 nm; DyLight, excitation 490–505 nm and emission 515–525 nm).

Images of ACTH and β-end immunoreactivities were captured on a Confocal Laser Scanning microscope (TCS SP5, Leica Microsystems CMS GmbH, Germany) using the following laser excitation lines: DyLight488 493 nm, and Cy3 543 nm. These filter combinations resulted in negligible cross talk between individual fluorochrome signals. All imaging was done in sequential scan mode to exclude cross-bleeding between different channels. Images were adjusted for contrast and brightness using Adobe Photoshop CS4.

### MD Simulation

All atom MD simulation is proven to be an important tool to understand the protein folding and protein aggregation *in silico* [20], [21], [22], [23], [24]. We performed all atom MD simulations to study the inter-peptide interactions between ACTH and β-end. We used the MD software package NAMD [25] and visualization tool VMD [26] in order to carry out the experimental procedures. The initial structures of ACTH and β-end for the simulation were constructed using the peptide builder tool in the molecular modeling and bioinformatics software package VEGA ZZ [27]. The coordinates were then subjected to intensive energy minimization using the conjugate-gradient algorithm for 15,000 steps. We performed MD simulation on the ACTH and β-end monomers in order to characterize structural fluctuations and to obtain a reliable starting structure for our simulation. Three sets of simulations were performed for 20 ns, each involving the study of interactions between four peptides placed in a solvent box: i) two molecules each of ACTH and β-end, ii) four molecules of ACTH, iii) four molecules of β-end. Identical simulation protocols were used for all simulation systems. The initial positions of the peptides within the simulation box were such that the orientations were random and the closest point between any two peptides in the simulation box was not less than 15 Å. The system was first energy minimized using conjugate gradient algorithm for 15,000 steps at an absolute zero temperature. The system was then linearly heated from 0 K to 310 K using temperature rescaling. Once the slow heating procedure was performed, the system was then equilibrated at 310 K for another 300 ps using reassigned velocities at every step. After the energy-minimization, linear heating, and equilibration steps, the system was used for the “production run” or “dynamic run” of 20 ns. In each of the 3 simulations, we used an integration time step of 2 fs. The van der Waals interaction cutoff used was 9 Å and the electrostatic interaction cutoff was 12 Å. The density of the water box was adjusted using NPT equilibration to ∼0.99 g/cm^3^. The size of the water box was dependant on the initial orientation and conformation of the peptides. The ACTH, β-end and ACTH-β-end combined systems were solvated in a simulation box of volume 1749763 Å^3^, 1063315 Å^3^ and 1394820 Å^3^ respectively.

## Results

### ACTH is natively unstructured peptide, non-aggregating and non-amyloidogenic

**Figure 1:**
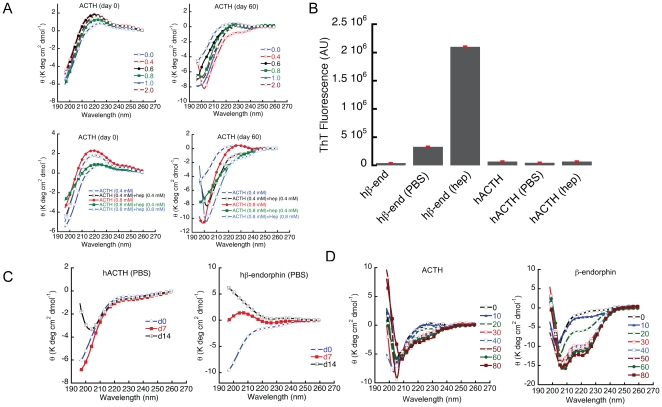
Structural transition and amyloid aggregation of ACTH and β-end. A) 2 mg/ml ACTH in presence of different concentrations of heparin were incubated for two months. Insignificant structural changes are observed at day 0 (top panel, left) and after two months (top panel, right) of incubation at 37°C with slight rotation at pH 5.5, suggesting that ACTH is highly soluble and non-amyloidogenic. Different symbols represent concentrations of heparin in mM. Similar experiments were also performed with various ACTH and heparin concentration ratios for two months (lower panel). No significant structural transformations were observed at day 0 (left, lower panel) and after two months of incubation (right, lower panel). B) ThT binding of ACTH and β-end samples incubated for two weeks either in PBS or in 5% D-mannitol at 37°C with slight rotation. Only β-end was able to bind ThT significantly, both in PBS and in 5% D-mannitol, 0.4 M heparin suggesting β-end is amyloidogenic. C) Conformational transition of 2 mg/ml ACTH (left) and β-end (right) incubated in PBS, pH 7.4, 0.01% sodium azide for two weeks. ACTH did not show significant conformational transition whereas β-end showed random coil to β-sheet transition after two weeks of incubation. D) TFE induced structural transition of ACTH and β-end. ACTH is unable to change its conformation even in presence of 80% TFE. Whereas, β-end changes its conformation from random coil to helix in presence of TFE concentration (>20% TFE v/v). Each symbol represents different concentrations of TFE (v/v).

**Figure 2:**
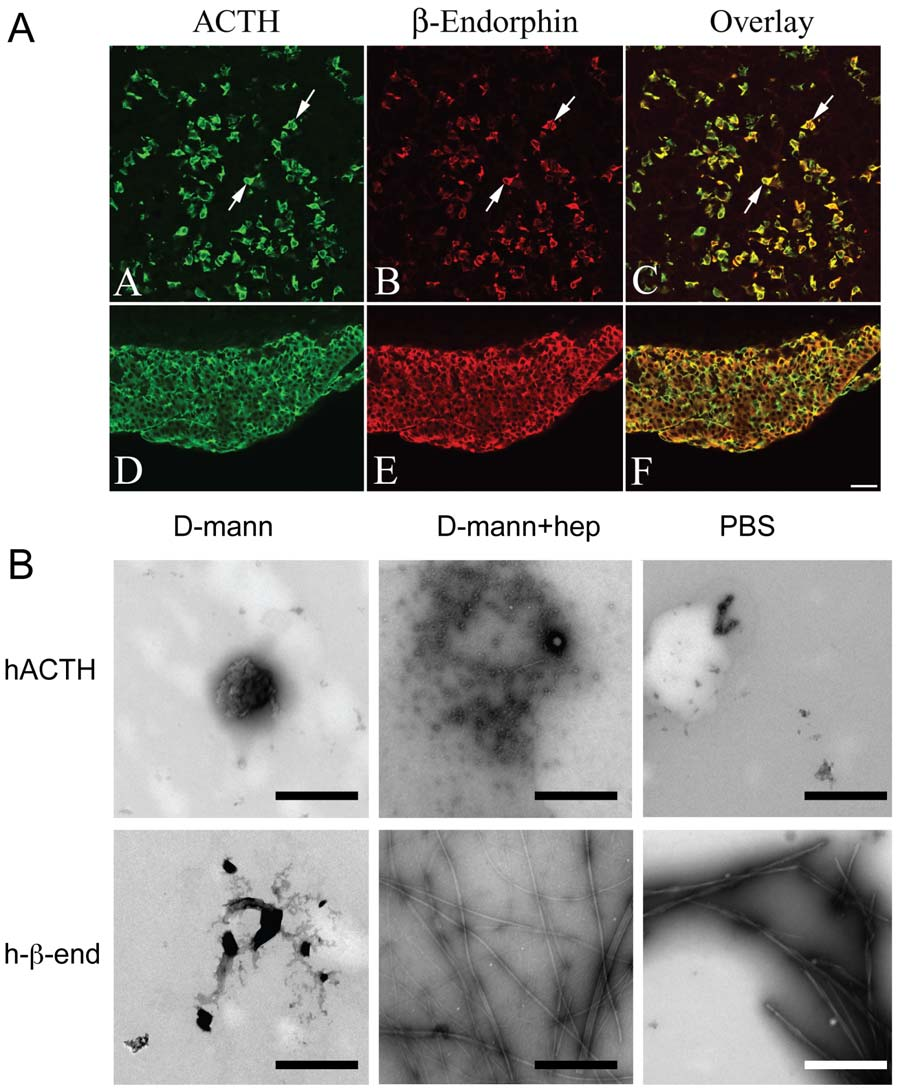
Aggregation and colocalization of ACTH and β-end. A) Fluorescence photomicrographs of the anterior (A–C) and intermediate (D–F) lobes of the pituitary gland showing ACTH (A, D), β-end (B, E), and ACTH-β-end colocalized (C, F) cells. Note that presence of several double-labeled cells (arrows) in anterior as well as intermediate lobes. Scale bar = 50 µm. B) EM images of ACTH and β-end samples incubated for 14 days. The aggregations of the hormones were followed at 37°C at a concentration of 2 mg/ml in the presence of 0.4 mM LMW heparin in 5% D-mannitol (pH 5.5) and PBS under slight agitation. TEM of negative stained samples was performed. Scale bars, 500 nm.

Previous studies of hACTH, rACTH and pACTH have suggested that none of the ACTHs self aggregated into amyloid-like fibrils *in vitro* [13]. However, ACTH co-aggregated with β-end *into* amyloid-like structure. Detailed studies were carried out to illustrate the self-aggregating behavior of hACTH at varying concentrations and also in presence of varying amounts of GAGs (heparin) in 5% D-mannitol, pH 5.5, 0.01% sodium azide (Figures 1A and B). Amyloid formations were monitored by CD, ThT binding and EM. CD studies indicated that ACTH did not undergo any structural transition even after two months of incubation (Figure 1A) under different conditions. Moreover, negligible ThT fluorescence of the two months incubated samples (Figure 1B) and absence of any amyloid-like morphology under EM suggest that ACTH is non-amyloidogenic (Figure 2B). We also examined whether a buffer with high salt concentration such as PBS might modulate the aggregation and amyloid formation of ACTH and β-end. The aggregation data in PBS, pH 7.4, 0.01% sodium azide suggested that β-end formed amyloid-like fibrils in PBS after two weeks of incubation (Figures 1B, 1C and 2B) as it showed random coil (RC) to β-sheet structural transition in CD, high ThT binding and appearance of fibrillar morphology under EM; whereas ACTH did not show any amyloid-like fibrils in identical experimental conditions. A decrease in CD signal at wavelength ∼198 nm was observed for ACTH after two weeks of incubation in PBS, indicating subtle change in secondary structure. However, CD signal in the wavelength range of 208–222 was mostly unchanged during incubation suggesting that major secondary structural transition was absent. To further evaluate whether ACTH has any intrinsic structural propensity, we initiated the experiment with TFE. TFE is known to destabilize hydrophobic interactions within the polypeptide chain and stabilizes local hydrogen bonds between residues close in the amino acid sequence [28]. It has long been suggested that TFE could induce helices in peptide/proteins in solution. To study the effect of TFE on secondary structures of ACTH and β-end, we performed CD experiments of 25 µM each of ACTH and β-end in PBS, pH 7.4 with increasing concentrations of TFE. The data suggested that ACTH is unable to change to any helical structure up to 80% of TFE concentration (v/v). Interestingly, β-end showed increased helicity in a TFE concentration dependent manner (Figure 1D). These results further suggest that ACTH does not possess any intrinsic tendency for structural change and/or aggregation by its own, but might change its structure in presence of β-end.

### ACTH and β-end are colocalized *in vivo*

Since ACTH and β-end are processed from the same prohormone (pro-opiomelanocortin) and secreted together via regulated secretory pathway [29], we hypothesized that ACTH might need the amyloid-forming β-end as an aggregation partner for its storage in secretory granules. The previous *in vitro* and immunofluorescence studies with AtT20 cells are consistent with this hypothesis [13]. Double labelling immunofluorescence study was performed using rat pituitary tissue to explore the anatomical substrates in the anterior pituitary where ACTH and β-end colocalize. We observed that most of the ACTHs are co-localized with β-end both in anterior and intermediate lobes of pituitary gland (Figure 2A).

### ACTH is unable to self-associate *in silico*

**Figure 3:**
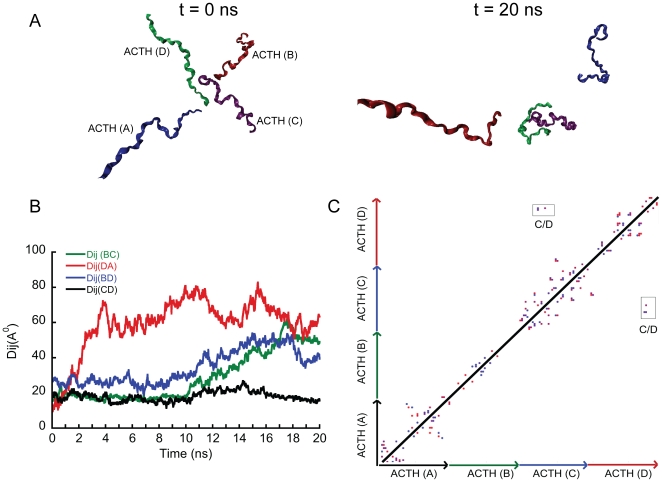
MD simulation of ACTH system. A) Snapshots at the left-hand side indicating initial state (t = 0 ns) and at the right-hand side indicating final state (t = 20 ns) of the simulation. B) Plot of distances between center of masses (Dij) of peptides within the ACTH simulation against time showing large interpeptide distances during simulation. C) Contact map at t = 20 ns snapshot showing residues in contact. Negligible interpeptide contacts are observed suggesting that peptides are not self-assembling *in silico*.

To understand the interpeptide interactions of ACTH *in silico*, all atom MD simulation was performed. Snapshots of initial and final configurations of the system are shown in Figure 3A whereas the detailed time progression is shown in Figure S1. From the radius of gyration (Rg) and root mean square deviation (RMSD) (Figures S2A and S2B) data, we observe structural fluctuations of the peptides for the entire duration of the simulation. The snapshots as well as the inter-peptide distances (Dij) plot (Figure 3B) indicate that major intermolecular contacts between ACTHs were absent during the 20 ns simulation. The distance between center of masses of the ACTH peptides are gradually increasing during the simulation period indicating that ACTH molecules have very little tendency to interact. Although subtle decrease in interpeptide distance between ACTH C and ACTH D were observed, distance between these two peptides remains ∼20 Å at the end of 20 ns simulation (Figure 3B). Consistent with interpeptide distances, significant hydrogen-bonding tendency was also absent. There was intermittent hydrogen bonding tendency observed for the initial part of the simulation that disappeared at the later phase, suggesting negligible tendency for inter-peptide hydrogen bonding among ACTHs *in silico*. This is further supported by the contact map data shown in Figure 3C. The sparse contacts seen between peptides C and D at the final time step are transient in nature. The structural transition during simulation has also suggested that most of the amino acid residues of ACTH are reluctant to undergo any major secondary structural transition in the entire duration of the simulation, except for a few residues of ACTH A (Figure S2C). This is consistent with our *in vitro* experimental studies.

### Self-association and structural transition of β-end *in silico*

**Figure 4:**
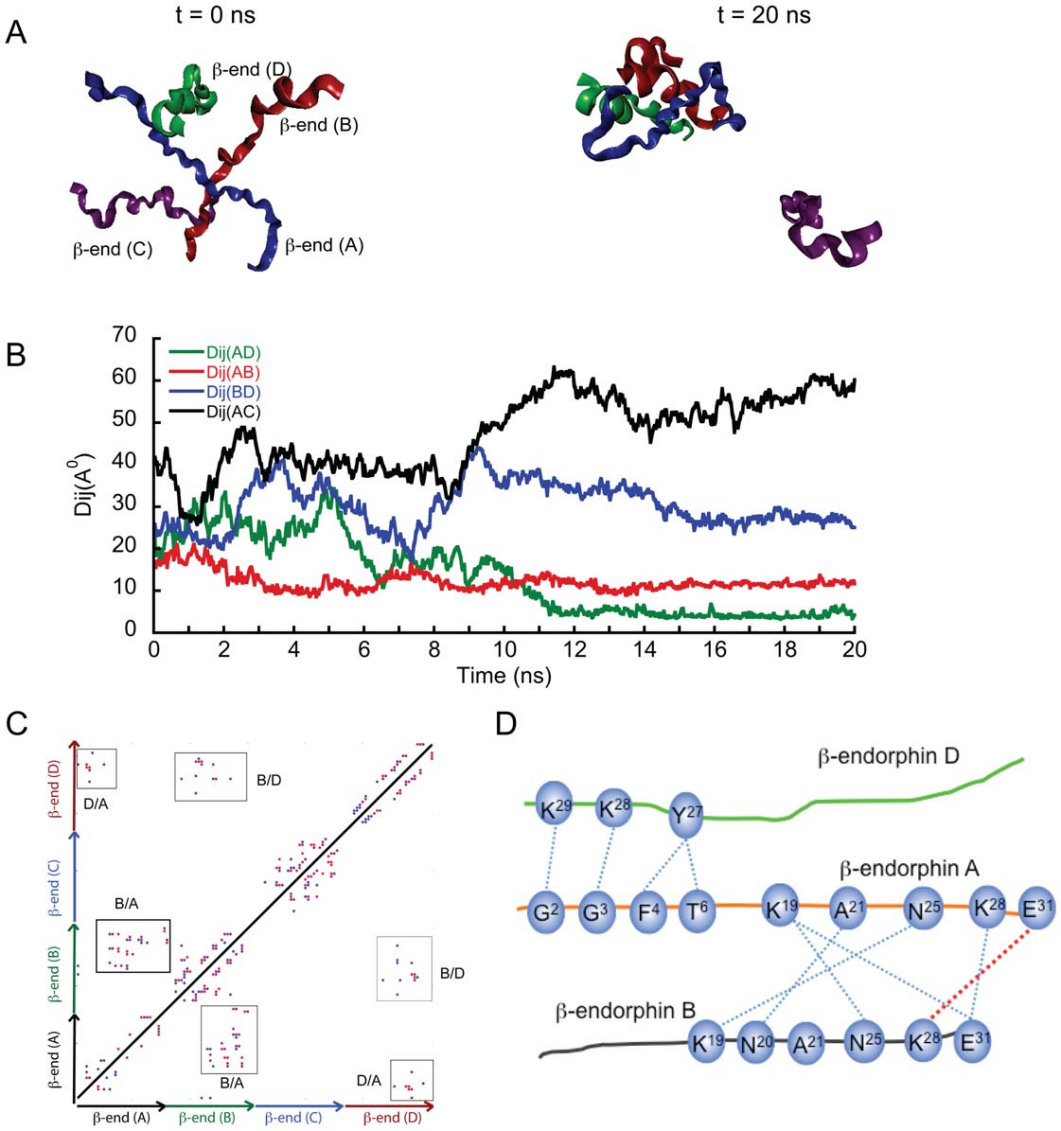
MD simulation of β-end system. All-atom MD simulation for the duration of 20 ns was performed in explicit solvent using four β-end. A) Snapshots indicating the initial (left) and final (right) states of the β-end system. B) Plot of distances between center of masses (Dij) of peptides within the β-end simulation against time. C) Contact map showing various residues in contact between peptides at t = 20 ns. Significant contacts are observed between amino acid residues in β-end A, β-end B and β-end D. D) A schematic depicting the various h-bonds observed between the peptides during the β-end simulation. The schematic showing C-terminus of β-end D is in contact with N-terminus of β-end A whereas C-terminus of β-end B is in contact with C-terminus β-end A. A salt bridge between E31 of β-end A and K28 of β-end B is represented by red dotted line.

Our *in vitro* studies suggested that β-end is able to undergo secondary structural transition from random coil to helix in presence of TFE and also shows amyloid formation in solution (Figures 1 and 2). To further understand the nature of the interaction, we performed *in silico* study using four β-end in a simulation box similar to the ACTH. The snapshots of initial and final configurations, shown in Figure 4A, indicate that three out of four β-end molecules interact in an intermolecular manner to form a trimeric assembly within the simulation box. The detailed progression of the assembly is shown in Figure S3. Rg and RMSD data (Figures S4A and S4B) for β-end show negligible fluctuation indicating higher stability, compared to that observed in ACTH. The separation of fourth β-end molecule from the trimer occurs at ∼10 ns of simulation (Figure S3). When the distance between center of masses of the β-end molecules were plotted against time (Figure 4B), there was a marked decrease in interpeptide distance observed for β-end A and β-end B (Dij AB), β-end A and β-end D (Dij AD). Similar trends are also observed to some extent with β-end B and β-end D. In contrast, an increase in separation was observed between β-end C and the trimeric assembly. The contact map at t = 20 ns suggests that significant interpeptide contacts have devoloped between β-ends A/B, A/D and B/D (Figure 4C), whereas no contacts were observed between β-end C and any other β-ends. The secondary structural transition during the simulation showed an increase in helical propensity in the β-end D (Figure S4C) whereas, signatures of β-strand propensity were only observed in β-end A of the trimeric assembly. The detailed examination of the hydrogen bonding (h-bonding) pattern within the trimer (depicted schematically in Figure 4D) suggests that C-terminus of β-end D was interacting with N-terminus of β-end A, whereas C-terminus of β-end A was interacting with C-terminus of β-end B. Only those hydrogen bonds, which were observed for more than 50% of the duration of the simulation, were considered for the schematic depiction. These interactions (Table S2) are mainly mediated by intermolecular h-bonding either through mainchain-mainchain or mainchain-sidechain or sidechain-sidechain interactions. Furthermore, a stable salt bridge was observed between Glu^31^ of β-end A and Lys^28^ of β-end B that existed for most of the simulation time.

### ACTH and β-end coaggregation *in silico*

**Figure 5:**
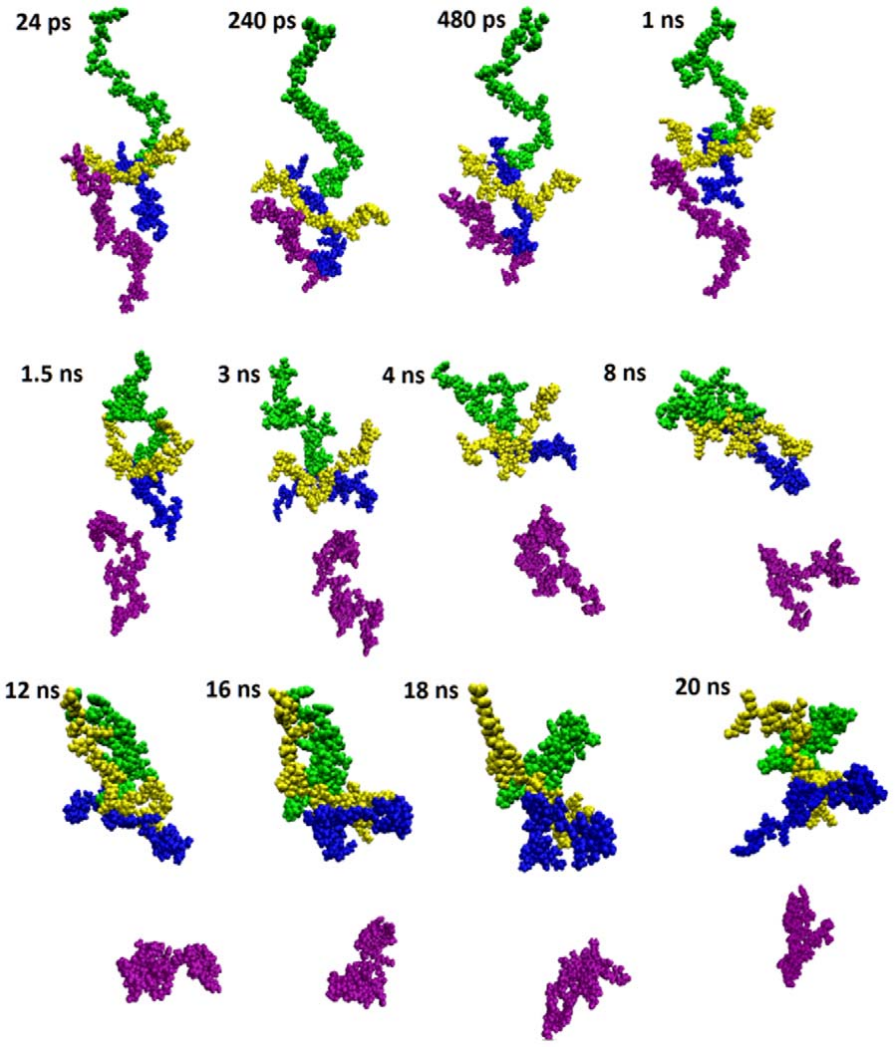
Snapshots at various time steps of ACTH-β-end simulation system showing hetero-oligomerization. The hetero-trimer consisting of two β-end and one ACTH is stable up to end of 20 ns simulation. β-end A, β-end B, ACTH C and ACTH D are represented by blue, yellow, green and purple color, respectively.

**Figure 6:**
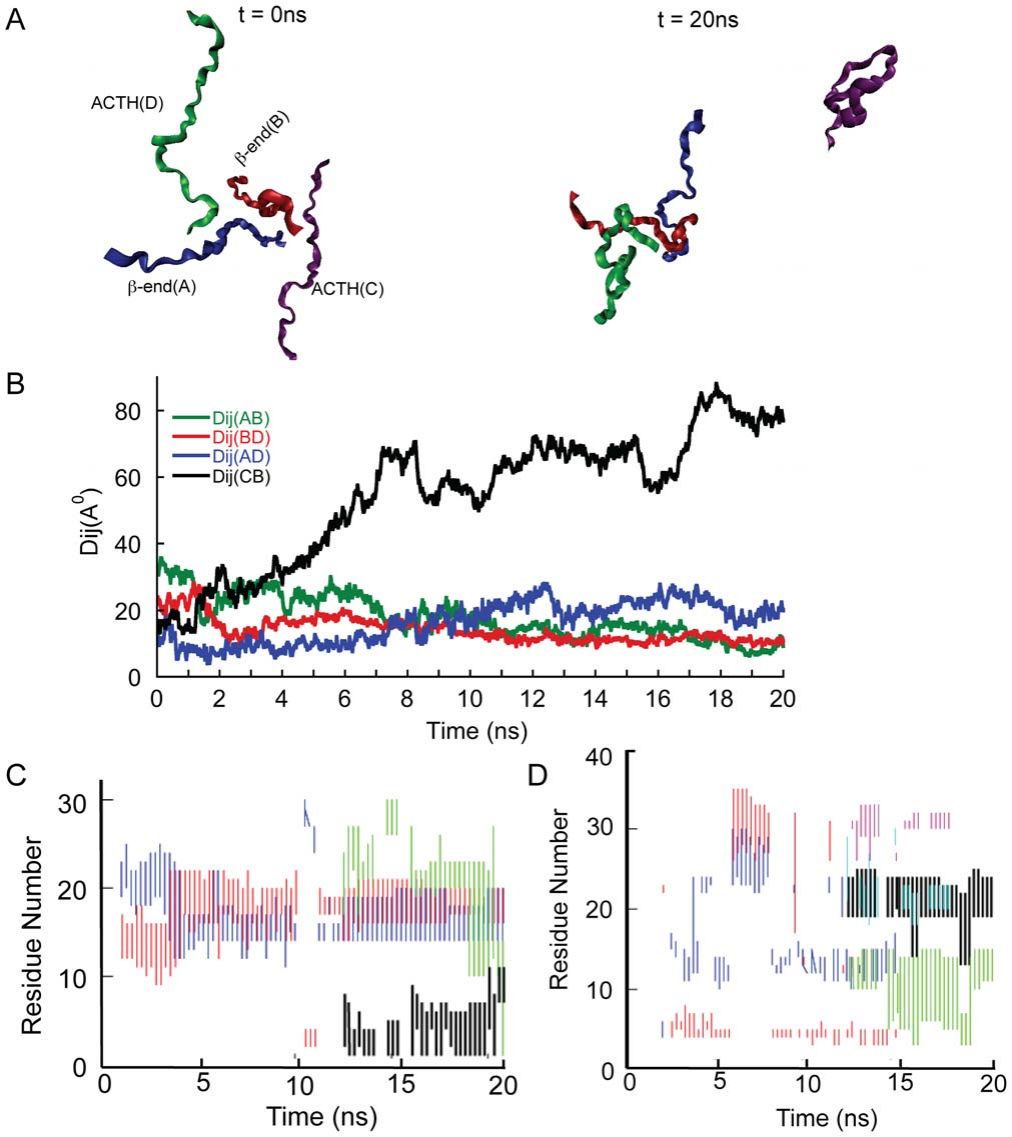
MD simulation of ACTH-β-end system. All-atom MD simulation for the duration of 20 ns was performed in explicit solvent using two ACTH and two β-end. **A**) Snapshots at the left-hand side indicating the initial configuration (t = 0 ns) and at right-hand side indicating the final configuration (t = 20 ns). **B**) Plot of distances between center of masses (Dij) of peptides against time indicating close proximity of peptides A, B and D. **C**) Time progression of contact regions between β-end A and β-end B. At any given time, the regions indicated by red lines of β-end B is in contact with regions indicated by blue lines of β-end A. Similarly, the regions indicated by black lines of β-end B is in contact with regions indicated by green lines of β-end A. The first contact pair (red and blue) is seen for the entire duration of the simulation whereas the second contact pair (black and green) appears only towards end of the simulation. **D**) Time progression of contact regions between ACTH D and β-end B. At any given time, the regions indicated by red lines of ACTH D is in contact with regions indicated by blue lines of β-end B. Similarly, the regions indicated by black lines of β-end B is in contact with regions indicated by green lines of ACTH D. The first contact pair (red and blue) is seen for the initial part of the simulation whereas the second contact pair (green and black) appears only towards end of the simulation. Additional contacts are also observed for certain duration between the region of β-end B (aqua line) and ACTH D (pink line).

**Figure 7:**
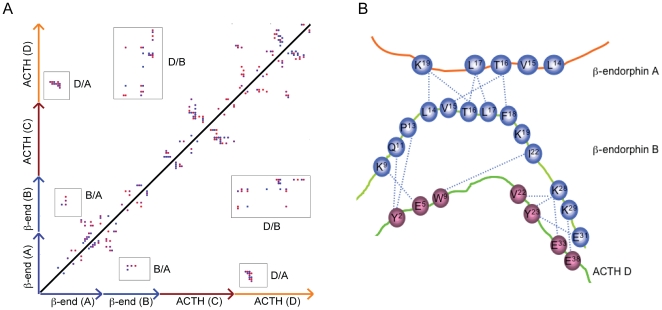
Analysis of the interpeptide interactions in ACTH-β-end system. A) Contact map showing various residues in contact within the ACTH-β-end simulation box at t = 20 ns. B) Schematic representation of the stable hydrogen bonds observed between the peptides during the simulation.

The *in vitro* and *in vivo* data suggested that ACTH and β-end co-aggregate to form amyloids, and co-localize in AtT20 cells and in rat pituitary tissues. However, the nature of interactions between these two peptides is not yet clear. To understand the early stages of coaggregation between ACTH and β-end, we performed MD simulation with two ACTH and two β-end in explicit solvent. As seen in the snapshots of the configurations, two β-end molecules get associated with one ACTH molecule to form a mixed trimeric assembly (Figures 5 and 6A). Rg and RMSD (Figures S5A and S5B) indicate that the system has approached a stable steady state towards the end of the simulation. Interestingly, ACTH C, which is not a part of the trimeric assembly, shows significantly higher fluctuations compared to the other peptides in the simulation box. The distance (Dij) vs time plot (Figure 6B) shows the separation of ACTH C from the peptides within the trimeric assembly and the proximity of the β-end A, β-end B and ACTH D. In order to assess the stability of various interactions, we analyzed inter-peptide contacts at various time steps of the simulation (Figures 6C and 6D). The data suggests that the approximate regions of Leu^14^-Asn^20^ of the two β-end molecules (A and B) are in contact for a prolonged duration of the simulation (Figure 6C). Additional contacts have also been observed between the residues near the C-Terminus (Ile^20^-Ala^26^) of β-end A and those at the N-terminus (Gly^3^-Ser^7^) of β-end B, towards the end of the simulation. This observation indicates that the orientation of the two β-end molecules that were initially at random, assumes an anti-parallel orientation towards the later part of the simulation. The contact progression between ACTH D and β-end B also shows similar stable contacts throughout the simulation (Figure 6D). Initial contacts were developed in the regions of (Asn^25^-Gly^30^) of β-end B and (Glu^28^-Phe^35^) of ACTH D. However, at later time steps, newer contacts were observed between the regions of (Lys^19^-Asn^25^) of β-end B and the region (Phe^7^-Lys^15^) of the ACTH D molecules (Figure 6D). The contact map at t = 20 ns (Figure 7A) shows that significant contacts exist between β-end A and B, and between β-end B and ACTH D. Occasional sparse contacts were also observed between ACTH D and β-end A. We also performed a detailed analysis of the h-bonding pattern between the peptides of the trimeric assembly. Those hydrogen bonds that are present for more than 50% of the total duration of the simulation (Table S3) are considered as “stable” hydrogen bonds, and are schematically depicted in Figure 7B. As shown in the figure, the β-end B is sandwiched between β-end A and ACTH D through h-bonding. The middle part of the β-end B is interacting with middle part of the β-end A in an anti-parallel fashion, while N- and C- termini of β-end B is interacting with N- and C- termini of the ACTH D in a parallel fashion. Importantly, the numbers of the stable hydrogen bonds are significantly more in this mixed system compared to the β-end-only system described earlier, suggesting that the mixed system is more stable. The plot of secondary structure progression of each of the peptides (Figure S5C) suggest an intermittent tendency to adopt the β-strand conformation at the region of Val^15^-Phe^18^ of both the interacting β-end (A and B). Additionally, some occasional β-strand tendency is observed in the regions Phe^4^-Ser^6^ of the β-end B that is in tight contact with ACTH D. The regions of β-end A and B showing β-strand tendency during the course of the simulation also coincide with the β-aggregation region, predicted by the TANGO algorithm [30]. Interestingly ACTH D, which is part of the trimer, also showed some β-strand tendency compared to the non-interacting ACTH C. The region between residues Pro^24^-Asp^29^ of ACTH D shows the maximum β-strand tendency and this is correspondingly the region, which is in contact with β-end B.

### N-terminal deletion of β-end affects the aggregation and amyloid formation tendency in β-end

**Figure 8:**
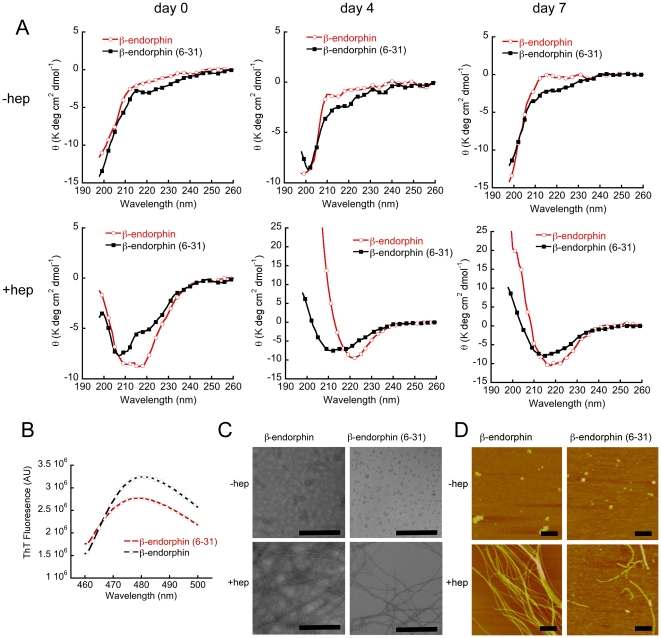
Effect of N-terminal deletion on β-end aggregation. A) CD spectra of full-length β-end and β-end (6–31) at 2 mg/ml concentration in 5% D-mannitol, 0.01% sodium azide, pH 5.5 in presence and absence of 400 µM of LMW heparin. In the absence of heparin, both peptides remain mostly unstructured up to one week of incubation at 37°C. In presence of heparin, the full-length β-end showing structural transition from α-helix at d0 to mostly β-sheet at d4; while the β-end (6–31), which was in a mixed conformation of random coil and helix at d0, showing mostly α-helical conformation at d4. Both peptides show mostly β-sheet conformation at d7. (B) The ThT fluorescence study of one week incubated sample in presence of heparin showing less ThT binding of β-end (6–31) compared to full-length β-end. C) EM studies of 7 days incubated samples showing numerous amyloid-like fibrils for full-length β-end and fewer fibrils for β-end (6–31) in presence of heparin. Any amyloid-like fibrils are not observed in absence of heparin for both the peptides. D) AFM images of the full-length β-end and β-end (6–31) in presence and absence of heparin after a 7-day incubation showing more number of fibrils in case of the full length β-end sample as compared to the β-end (6–31) sample. Scale bars are of 500 nm.

From our *in silico* simulation study of β-end and β-end/ACTH system, we observed that many intermolecular interactions (h-bonds and salt bridges; see Table S2 and S3) are responsible for the early oligomerization event of these peptide self-assemblies. For example, the Figure 4 shows that N-terminal Gly^2^-Thr^6^ amino acid residues of β-end A are interacting with C-terminal Tyr^27^-Lys^29^ amino acid residues of β-end D. To validate these findings, we performed *in vitro* aggregation experiments of full-length β-end and N-terminally truncated β-end (β-end (6–31) using CD, ThT, EM and AFM. In our CD studies, we observed that both β-end and β-end (6–31) showed mostly unstructured nature during one week of incubation in 5% d-mannitol. However, in presence of LMW heparin, β-end showed mostly α-helical conformation at day 0 and transformed to β-sheet at day 4 (Figure 8A); while β-end (6–31) showed mixture of random coil and helix at day 0, which transformed to a predominantly α-helical conformation at day 4. The data suggest a retardation of conformational transition to β-sheet in β-end (6–31). The ThT fluorescence study of one week incubated samples in presence of heparin showed less ThT binding of β-end (6–31) compared to full-length β-end (Figure 8B). The EM data (Figure 8C) of 7 days incubated samples showed that numerous amyloid-like fibrils were formed by β-end, whereas very thin and fewer fibrils were formed by β-end (6–31). In the absence of heparin, both full-length β-end and β-end (6–31) did not show any amyloid fibril formation, rather only small oligomers were observed. AFM study of 7 days incubated sample of β-end and β-end (6–31) also showed similar results (Figure 8D). The combined data of CD, morphological study by EM, AFM and ThT fluorescence suggest that truncation of 1–5 amino acid residues result in slower aggregation kinetics and reduced amyloid formation compared to full-length β-end.

## Discussion

It was suggested that co-aggregation might be an essential mechanism for nonaggregating peptide/proteins to be secreted via regulated secretory pathways [13], [31]. Previous *in vitro* studies using exocrine enzymes that are stored in secretory granules have suggested that many exocrine enzymes are able to aggregate under granule-relevant conditions *in vitro*. However, amylase, an exocrine enzyme, does not show homotypic aggregation under similar conditions [31], [32]. Interestingly, this enzyme aggregates in presence of exocrine granular content, suggesting that heterotypic aggregation might play an important role for concentrating and sorting of this enzyme in the secretory granules. The previous studies have suggested that many peptide hormones such as ACTHs (rACTH, pACTH and hACTH) and ghrelins do not aggregate to form amyloids *in vitro* [13]. However, they aggregate into amyloids in presence of their appropriate partner peptide further suggesting that heterotypic aggregation might also be responsible for their secretory granule formation in endocrine cells.

In our study, ACTH did not aggregate either with varying peptide/heparin ratio or in PBS even after two months of incubation. This clearly indicates that ACTH is a highly soluble peptide hormone and is incapable for aggregation and amyloid formation. In contrast, β-end formed amyloids both in presence of heparin (in D-mannitol) and in PBS. The TFE titration results indicated that ACTH is also incapable of undergoing structural transition even in presence of 80% TFE (v/v) whereas β-end showed helical structure when titrated with varying concentration of TFE. All these studies clearly suggest that ACTH might require aggregation partner for its structural transition and amyloid formation. This suggestion is further strengthened by the fact that ACTH and β-end are largely colocalized in the pituitary cells [33]. Using alternate sections, concomitant storage of ACTH and β-end immunoreactivity has been shown in the secretory granules of anterior pituitary [34]. We observed that in the anterior and intermediate lobes of the pituitary, ACTH and β-end are present in the same cells.

In conjunction with our *in vitro* and *in vivo* data, MD simulation studies of the three systems (ACTH+β-end, ACTH-only, and β-end-only) also clearly show that ACTH cannot undergo homotypic association; they rather show a higher tendency to associate with β-end suggesting a heterotypic mechanism. The ACTH-β-end mixed trimeric system showed more number of interpetide contacts as compared to the trimer of the β-end-only system suggesting that the former is the more stable system among the two. This is further supported by the interpeptide contacts observed during the 20 ns simulation time. Our data also indicates that hydrophobic contacts might initiate both trimer and mixed trimer formation. As seen in the case of β-end A and β-end B of the mixed trimer, the region between the residues Leu^14^-Lys^19^ of β-end A and B, which is a part of the most hydrophobic region calculated by the Kyte Doolittle hydrophobicity scale [35], is involved in forming stable contacts. ACTH molecules on the other hand are lacking such a hydrophobic region, which might be needed for self-aggregation, as observed in case of β-end. The observation of more number of stable h-bonds in the mixed trimer system, when compared to the β-end-only trimer, suggest that ACTH increases the overall stability of the mixed trimer system. This type of heterotypic assembly, on one hand, might be favorable for ACTH to get packaged and stored; on the other hand, also advantageous for β-end as ACTH increases the number of stable h-bonds within the aggregate. The results suggest that the interaction between the peptides in the ACTH-β-end system might help to decrease the fluctuations, by providing an additional degree of stability to the system compared to β-end-only system. Interestingly, ACTH interacting with β-end showed some secondary structural transition suggesting that β-end is absolutely required for this structural transition. The previous *in vitro* data have also suggested that tryptophan residue in ACTH in the ACTH-β-end aggregates showed a blue shift in λ_max_ compared to the monomeric peptides, suggesting a change in microenvironment [13] during aggregation.

### Conclusion

Cross-seeding and hetero-aggregation are implicated in many human diseases and peptide hormone storage in secretory granules [13], [31], [36], [37]. Here, we attempted to understand coaggregation of ACTH-β-end using a wide variety of experimental and computational approaches. Our evidences strengthen the hypothesis that ACTH peptide does not have the ability to form aggregated structures; it needs the presence of β-end or similar interacting partner to get aggregated *in vitro* and *in silico*. While ACTH certainly gets benefited in the coaggregation with the β-end, it could also be a preferred mechanism for β-end to be condensed and packaged into secretory granules in a more stable manner. It is important to note that the number of peptides used in the simulation and the timescale of the simulation is limited, thereby the simulation does not access the timescale required for fibrillization. It is also possible that both β-end and ACTH/β-end system require equimolar amount of heparin (in accordance to *in vitro* study [13]) in the simulation box for significant structural transition of the peptides into β-sheet structure *in silico*. However, our simulations were aimed at studying the tendency of the peptides to form inter-peptide contacts at early stages of oligomerization, which might later initiate the secondary structure transition from random coil to β-strand as well as amyloid-like fibril formation. The delayed *in vitro* aggregation kinetics of truncated β-end (6–31) suggest that the N-terminal residues of β-end play an important role for its aggregation and amyloid formation, which is consistent with our *in silico* findings.

Our data thus not only provide possible regions for inter-peptide association and nature of the interactions but also helps to understand the mechanism of secretory granule biogenesis by ACTH and β-end.

## Supporting Information

Figure S1 **Snapshots showing time progression of the ACTH simulation.** Significant intermolecular association was not observed. However, weak interactions between ACTH C and ACTH D are evident at the end of 20 ns simulation. ACTH A, B, C and D are represented by blue, red, purple and green color, respectively.
(TIF)

Figure S2 **Structural fluctuations and secondary structure progression of ACTH system.** A) Time progression of the radius of gyration (Rg) of individual peptides within the ACTH system showing higher degree of fluctuation compared to ACTH-β-end system (see Figure S5). B) The fluctuation observed in the RMSD Vs time plot is also consistent with radius of gyration. C) Secondary structure progression of amino acid residues (N-terminus (top) to C-terminus (bottom)) of all peptides during the simulation. Only ACTH A and ACTH C showed some secondary structural transition from random coil to helix at certain regions. Other two ACTHs failed to show any secondary structural transition. White, green, blue, pink, yellow colors indicate random coil, turn, π-helices, α-helices, β-strand, respectively.
(TIF)

Figure S3 **Snapshots at various time steps of β-end simulation system showing oligomerization of β-end.** One of the β-end was separated out from the other three β-end that form trimeric assembly, which is stable up to 20 ns. β-end A, B, C and D are represented by blue, red, purple and green color, respectively.
(TIF)

Figure S4 **Structural fluctuations and secondary structure progression of β-end system.** A) Time progression of the radius of gyration (Rg) of individual peptides within the β-end system indicating the system attains stability towards the end of simulation. B) The RMSD Vs time plot of all peptides within the β-end system indicating the system has approached a steady state. C) Secondary structure progression of amino acid residues (N-terminus (top) to C-terminus (bottom)) of all peptides during the simulation. Only β-end C and β-end D showed some secondary structural transition from random coil to helix at certain regions. White, green, blue, pink, yellow colors indicate random coil, turn, π-helices, α-helices, β-strand, respectively. Occasional appearances of β-strand are seen in few residues of β-end A whereas β-end B failed to show any secondary structure transition during simulation.
(TIF)

Figure S5 **Structural fluctuations and secondary structure progression of ACTH-β-end system.** A) Time progression of the radius of gyration of individual peptides within the ACTH-β-end system indicating the system attains stability towards the end of simulation. B) The RMSD Vs time plot of all peptides within the ACTH-β-end system showing that peptides within the trimeric assembly attains steady state towards the end of simulation. C) Secondary structure progression of amino acid residues of all peptides (N-terminus (top) to C-terminus (bottom)) during the simulation. β-end A showed some secondary structural transition from random coil to helix at certain regions. Occasional appearances of β-strands are seen in few residues of β-end B and ACTH D both of which interact with each other to form a mixed trimer. White, green, blue, pink, yellow colors indicate random coil, turn, π-helices, α-helices, β-strand, respectively.
(TIF)

Table S1 **Amino acid sequences of hACTH and hβ-end.**
(DOC)

Table S2 **Major intermolecular interactions in β-end simulation.**
(DOC)

Table S3 **Major interactions between ACTH and β-end.**
(DOC)
