# Supplementary figures and images for: Molecular Interpretation of ACTH-β-Endorphin Coaggregation: Relevance to Secretory Granule Biogenesis
Source: PLoS One. 2012 Mar 5;7(3):e31924. doi: 10.1371/journal.pone.0031924 (PMC3293876; doi:10.1371/journal.pone.0031924)

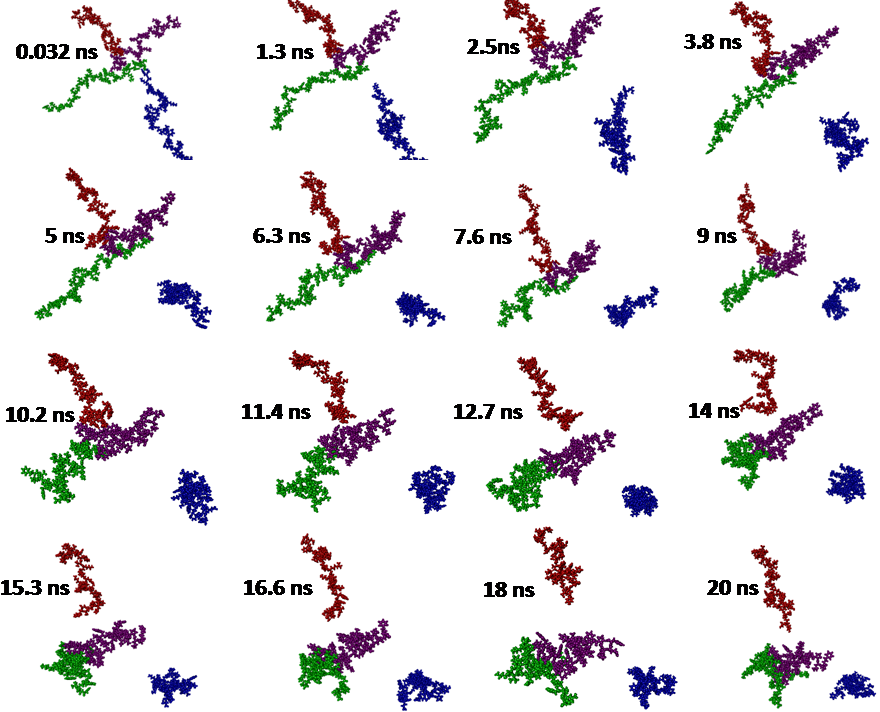

Supplement: Figure S1 — Snapshots showing time progression of the ACTH simulation. Significant intermolecular association was not observed. However, weak interactions between ACTH C and ACTH D are evident at the end of 20 ns simulation. ACTH A, B, C and D are represented by blue, red, purple and green color, respectively. (TIF) [file pone.0031924.s001.tif]

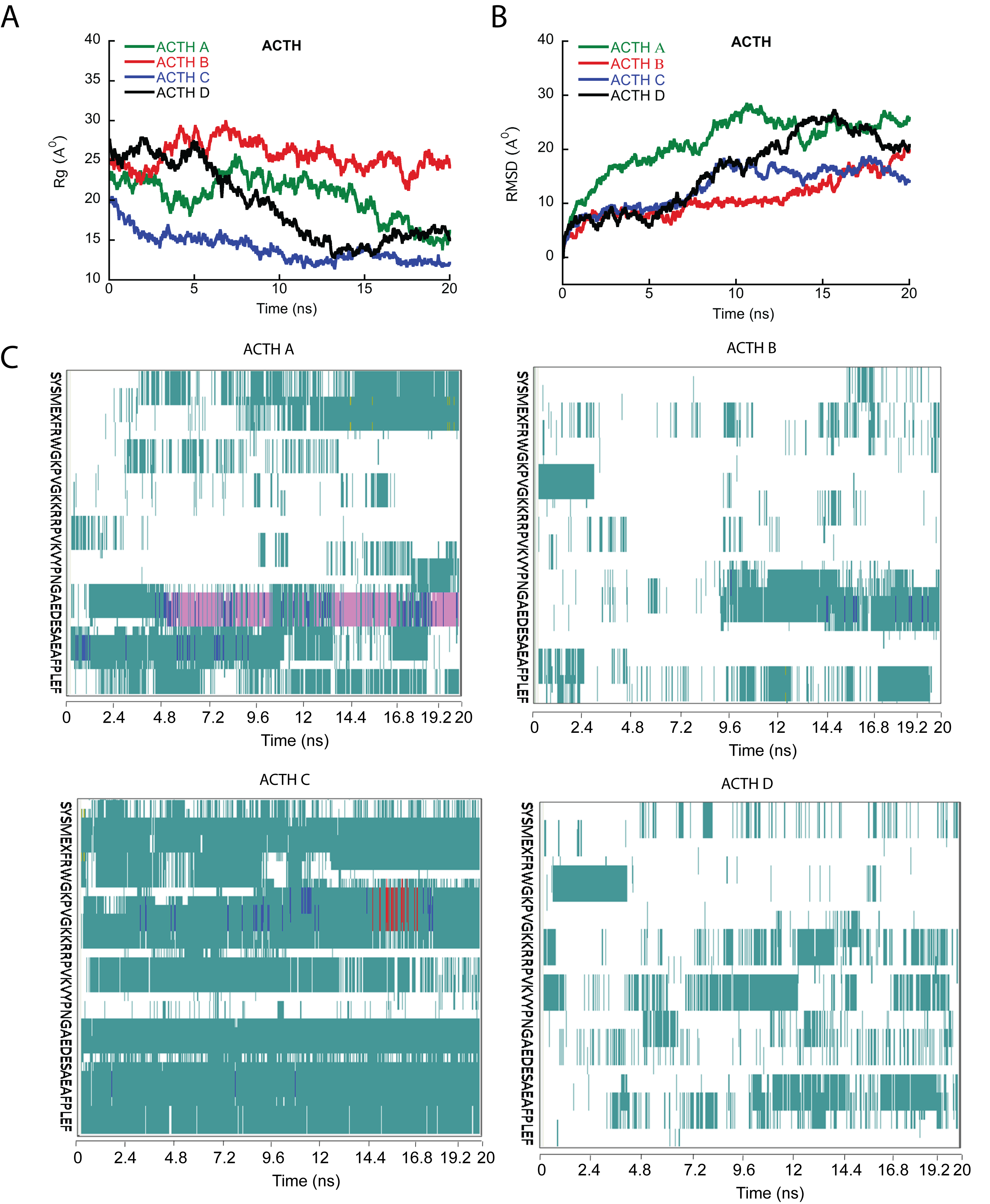

Supplement: Figure S2 — Structural fluctuations and secondary structure progression of ACTH system. A) Time progression of the radius of gyration (Rg) of individual peptides within the ACTH system showing higher degree of fluctuation compared to ACTH-β-end system (see Figure S5). B) The fluctuation observed in the RMSD Vs time plot is also consistent with radius of gyration. C) Secondary structure progression of amino acid residues (N-terminus (top) to C-terminus (bottom)) of all peptides during the simulation. Only ACTH A and ACTH C showed some secondary structural transition from random coil to helix at certain regions. Other two ACTHs failed to show any secondary structural transition. White, green, blue, pink, yellow colors indicate random coil, turn, π-helices, α-helices, β-strand, respectively. (TIF) [file pone.0031924.s002.tif]

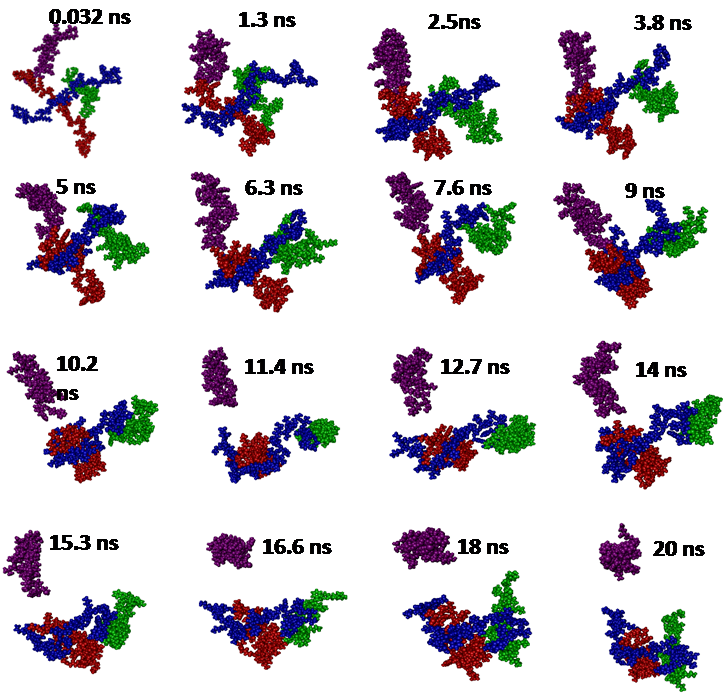

Supplement: Figure S3 — Snapshots at various time steps of β-end simulation system showing oligomerization of β-end. One of the β-end was separated out from the other three β-end that form trimeric assembly, which is stable up to 20 ns. β-end A, B, C and D are represented by blue, red, purple and green color, respectively. (TIF) [file pone.0031924.s003.tif]

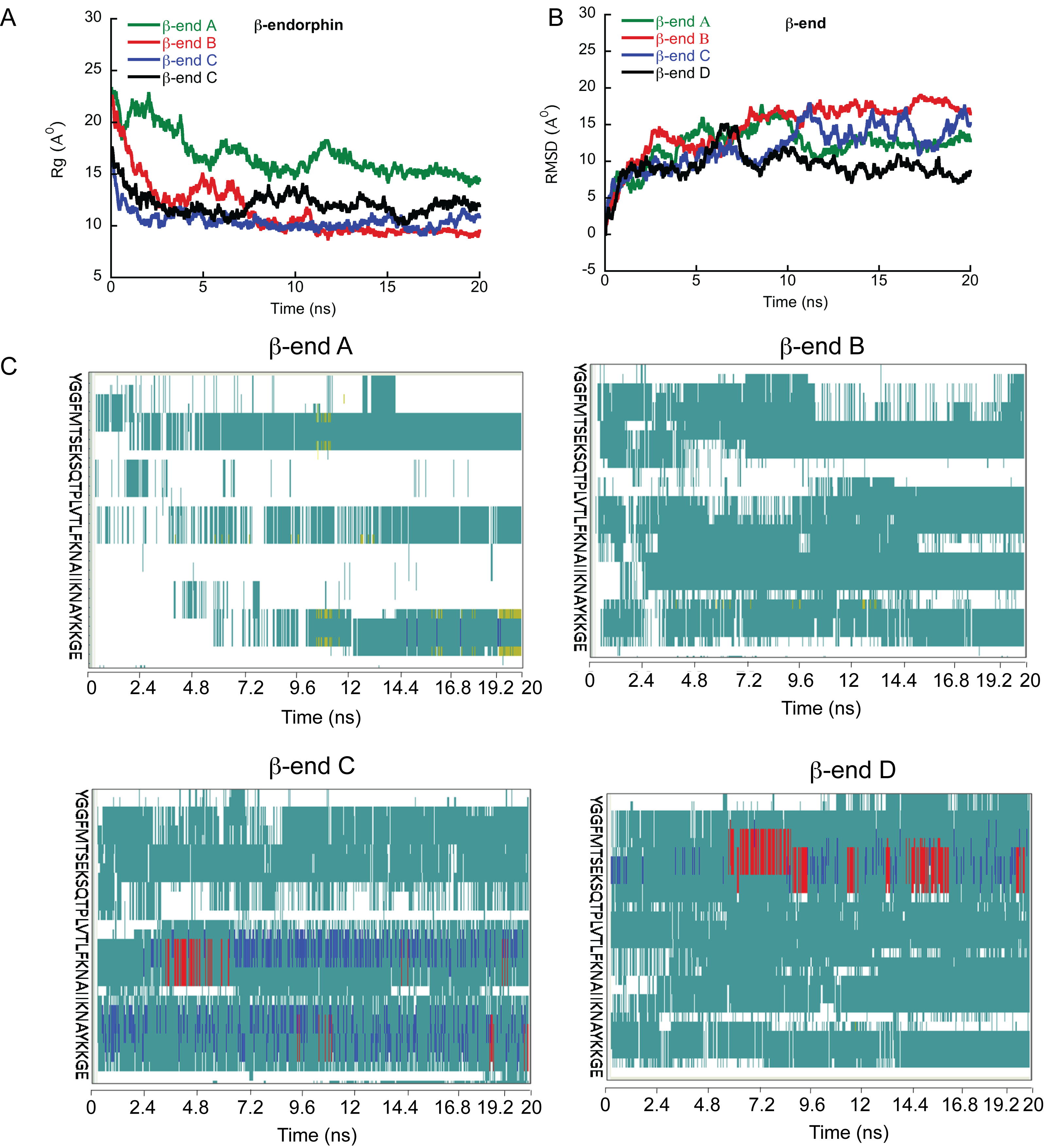

Supplement: Figure S4 — Structural fluctuations and secondary structure progression of β-end system. A) Time progression of the radius of gyration (Rg) of individual peptides within the β-end system indicating the system attains stability towards the end of simulation. B) The RMSD Vs time plot of all peptides within the β-end system indicating the system has approached a steady state. C) Secondary structure progression of amino acid residues (N-terminus (top) to C-terminus (bottom)) of all peptides during the simulation. Only β-end C and β-end D showed some secondary structural transition from random coil to helix at certain regions. White, green, blue, pink, yellow colors indicate random coil, turn, π-helices, α-helices, β-strand, respectively. Occasional appearances of β-strand are seen in few residues of β-end A whereas β-end B failed to show any secondary structure transition during simulation. (TIF) [file pone.0031924.s004.tif]

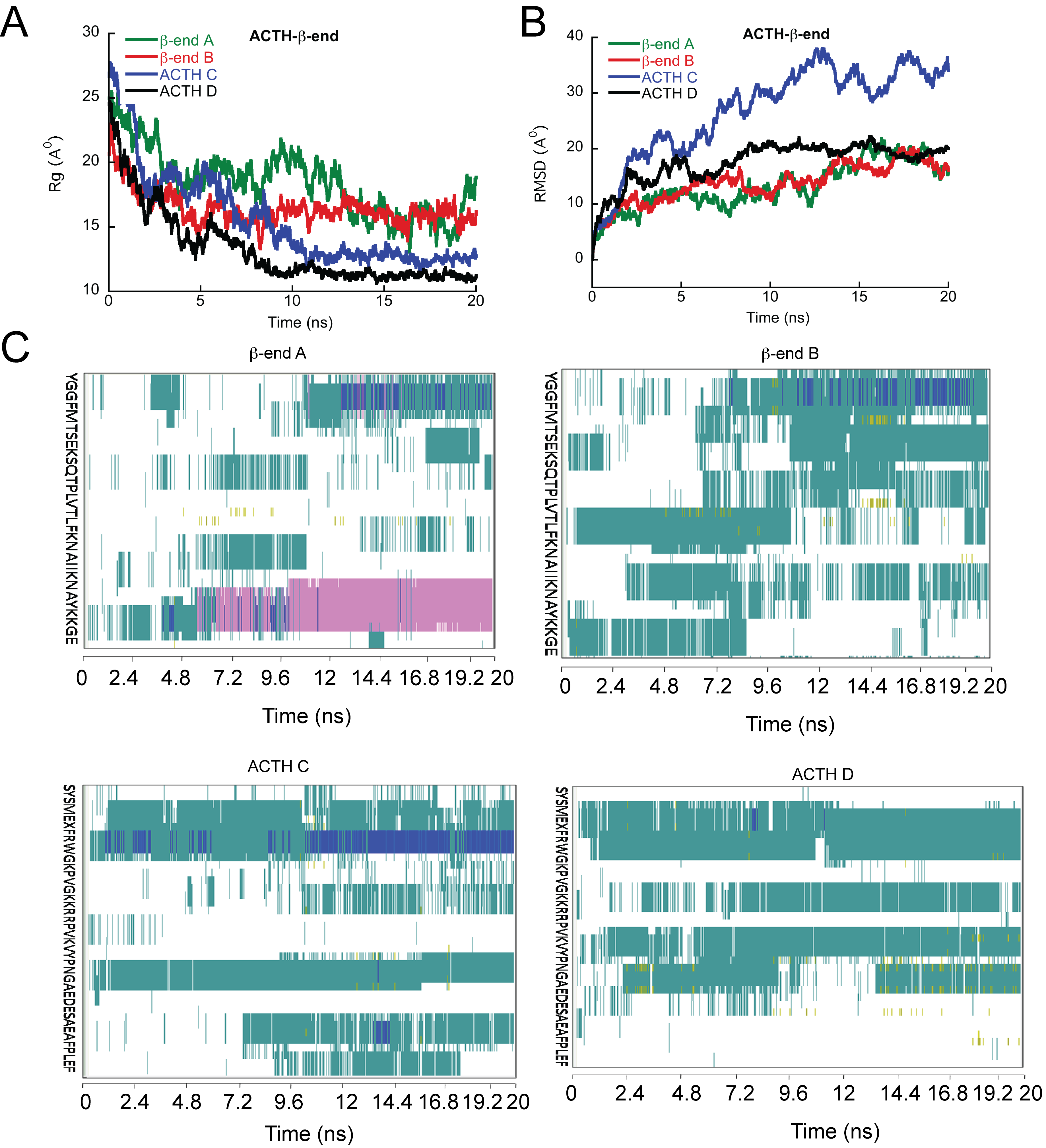

Supplement: Figure S5 — Structural fluctuations and secondary structure progression of ACTH-β-end system. A) Time progression of the radius of gyration of individual peptides within the ACTH-β-end system indicating the system attains stability towards the end of simulation. B) The RMSD Vs time plot of all peptides within the ACTH-β-end system showing that peptides within the trimeric assembly attains steady state towards the end of simulation. C) Secondary structure progression of amino acid residues of all peptides (N-terminus (top) to C-terminus (bottom)) during the simulation. β-end A showed some secondary structural transition from random coil to helix at certain regions. Occasional appearances of β-strands are seen in few residues of β-end B and ACTH D both of which interact with each other to form a mixed trimer. White, green, blue, pink, yellow colors indicate random coil, turn, π-helices, α-helices, β-strand, respectively. (TIF) [file pone.0031924.s005.tif]
