# Supplementary material for: Molecular Interpretation of ACTH-β-Endorphin Coaggregation: Relevance to Secretory Granule Biogenesis
Source: PLoS One. 2012 Mar 5;7(3):e31924. doi: 10.1371/journal.pone.0031924 (PMC3293876; doi:10.1371/journal.pone.0031924)
Supplement: Table S1 — Amino acid sequences of hACTH and hβ-end. (DOC) [file pone.0031924.s006.doc]

**Table S1.** **Amino acid sequences of hACTH and hβ-end.**

| Hormones | Amino acid sequences |
| --- | --- |
| hACTH | SYSMEHFRWG^10^ KPVGKKRRPV^20^ KVYPNGAEDE^30^ SAEAFPLEF^39^ |
| hβ-end | YGGFMTSEKS^10^ QTPLVTLFKN^20^ AIIKNAYKKG^30^ E^31^ |
