# Supplementary material for: Molecular Interpretation of ACTH-β-Endorphin Coaggregation: Relevance to Secretory Granule Biogenesis
Source: PLoS One. 2012 Mar 5;7(3):e31924. doi: 10.1371/journal.pone.0031924 (PMC3293876; doi:10.1371/journal.pone.0031924)
Supplement: Table S2 — Major intermolecular interactions in β-end simulation. (DOC) [file pone.0031924.s007.doc]

**Table S2:** **Major intermolecular interactions in β-end simulation.**

| Interaction of peptides | Residue involved | Interaction type |
| --- | --- | --- |
| β-end A and β-end B | Lys^19^–Asn^25^ | Main–Main^*^ |
|  | Lys^19^–Glu^31^ | Side–Side |
|  | Asn^25^–Lys^19^ | Side-Main |
|  | Ala^21^-Asn^20^ | Main – Side |
|  | Lys^28^–Glu^31^ | Side – Side |
|  | Glu^31^-Lys^28^ | Salt Bridge |
|  | | |
| β-end A and β-end D | Gly^3^–Lys^28^ | Side – Main |
|  | Gly^2^–Lys^29^ | Side – Main |
|  | Phe^4^–Tyr^27^ | Main – Main |
|  | Thr^6^–Lys^28^ | Side-Main |

^*^Main and side are representing main chain and side chain, respectively.
