# Supplementary material for: Molecular Interpretation of ACTH-β-Endorphin Coaggregation: Relevance to Secretory Granule Biogenesis
Source: PLoS One. 2012 Mar 5;7(3):e31924. doi: 10.1371/journal.pone.0031924 (PMC3293876; doi:10.1371/journal.pone.0031924)
Supplement: Table S3 — Major interactions between ACTH and β-end. (DOC) [file pone.0031924.s008.doc]

**Table S3:** **Major interactions between ACTH and β-end.**

| Interaction of peptides | Residue involved | Interaction type |
| --- | --- | --- |
| β-end A and β-end B | Lys^19^–Leu^14^ | Side – Main^*^ |
|  | Lys^19^–Thr^16^ | Side – Side |
|  | Leu^17^–Thr^16^ | Main – Main |
|  | Thr^16^–Val^15^ | Side – Main |
|  | Leu^17^–Leu^17^ | Main – Main |
|  | Thr^16^–Phe^18^ | Side – Main |
|  | | |
| β-end B and ACTH D | Lys^9^–Glu^5^ | Side – Side |
|  | Gln^11^–Tyr^2^ | Side – Main |
|  | Glu^31^-Tyr^23^ | Side – Side |
|  | Lys^28^–Tyr^23^ | Main – Side |
|  | Lys^28^-Glu^33^ | Side – Side |
|  | Lys^28^–Val^22^ | Side – Main |
|  | Pro^13^-Tyr^2^ | Side – Main |
|  | Lys^29^–Glu^38^ | Side – Side |
|  | Ile^22^–Trp^9^ | Main - Main |

^*^Main and side are representing main chain and side chain, respectively.
